# Differential Temporal and Spatial Progerin Expression during Closure of the Ductus Arteriosus in Neonates

**DOI:** 10.1371/journal.pone.0023975

**Published:** 2011-09-06

**Authors:** Regina Bökenkamp, Vered Raz, Andrea Venema, Marco C. DeRuiter, Conny van Munsteren, Michelle Olive, Elizabeth G. Nabel, Adriana C. Gittenberger-de Groot

**Affiliations:** 1 Department of Pediatric Cardiology, Leiden University Medical Center, Leiden, The Netherlands; 2 Department of Human Genetics, Leiden University Medical Center, Leiden, The Netherlands; 3 Department of Anatomy and Embryology, Leiden University Medical Center, Leiden, The Netherlands; 4 National Heart, Lung and Blood Institute, Bethesda, Maryland, United States of America; 5 Brigham and Women's Hospital, Boston, Massachusetts, United States of America; University of Giessen Lung Center, Germany

## Abstract

Closure of the ductus arteriosus (DA) at birth is essential for the transition from fetal to postnatal life. Before birth the DA bypasses the uninflated lungs by shunting blood from the pulmonary trunk into the systemic circulation. The molecular mechanism underlying DA closure and degeneration has not been fully elucidated, but is associated with apoptosis and cytolytic necrosis in the inner media and intima. We detected features of histology during DA degeneration that are comparable to Hutchinson Gilford Progeria syndrome and ageing. Immunohistochemistry on human fetal and neonatal DA, and aorta showed that lamin A/C was expressed in all layers of the vessel wall. As a novel finding we report that progerin, a splicing variant of lamin A/C was expressed almost selectively in the normal closing neonatal DA, from which we hypothesized that progerin is involved in DA closure. Progerin was detected in 16.2%±7.2 cells of the DA. Progerin-expressing cells were predominantly located in intima and inner media where cytolytic necrosis accompanied by apoptosis will develop. Concomitantly we found loss of α-smooth muscle actin as well as reduced lamin A/C expression compared to the fetal and non-closing DA. In cells of the adjacent aorta, that remains patent, progerin expression was only sporadically detected in 2.5%±1.5 of the cells. Data were substantiated by the detection of mRNA of progerin in the neonatal DA but not in the aorta, by PCR and sequencing analysis. The fetal DA and the non-closing persistent DA did not present with progerin expressing cells. Our analysis revealed that the spatiotemporal expression of lamin A/C and progerin in the neonatal DA was mutually exclusive. We suggest that activation of *LMNA* alternative splicing is involved in vascular remodeling in the circulatory system during normal neonatal DA closure.

## Introduction

**Figure 1:**
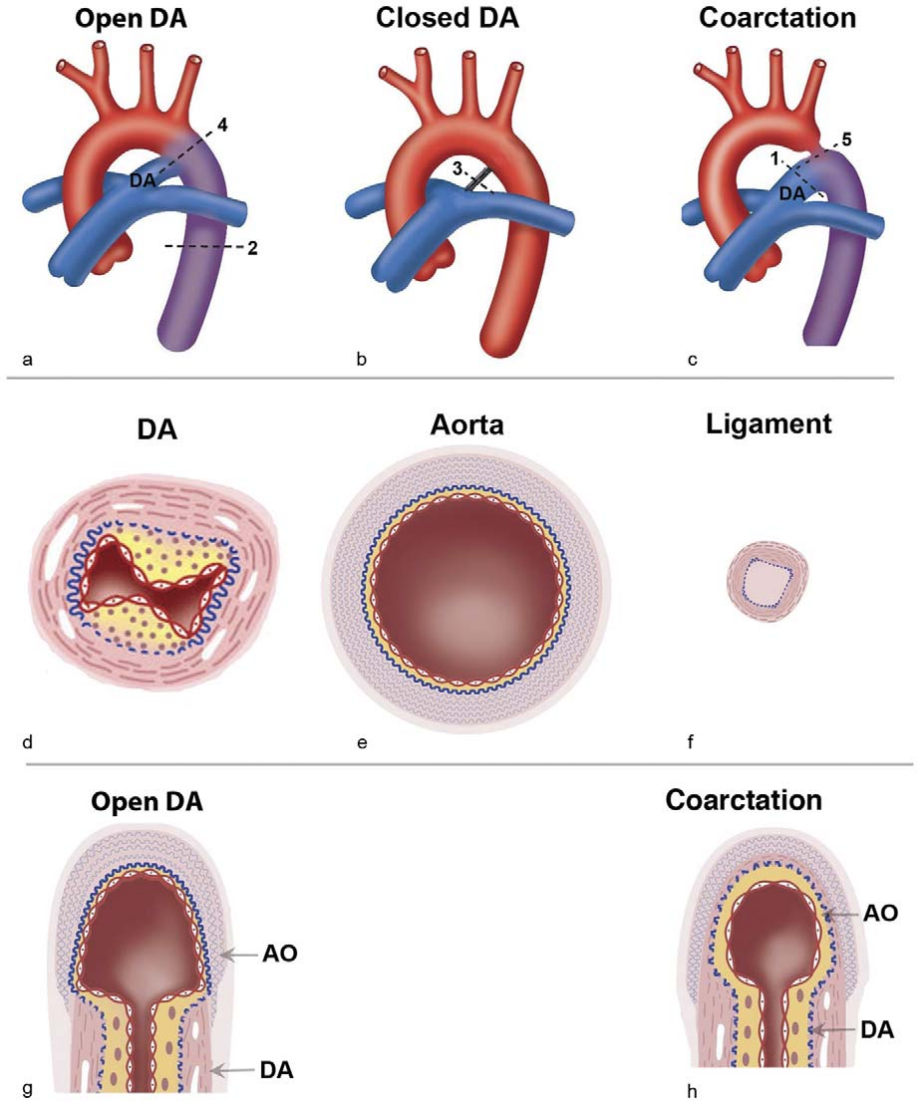
Schematic presentations of the great vessels. Anatomy of DA and the adjacent arteries. (a) A patent DA is found in the fetus before respiration starts. Oxygen-rich blood (in red) from the placenta passes though the aorta. Oxygen-poor blood from the fetus (in blue) passes the DA entering the descending aorta (in purple). (b) After the start of respiration the DA regresses into a ligamentum arteriosum. Aortic (red) and pulmonary (blue) circulation are disconnected. (c) In aortic coarctation, a left sided heart malformation, the DA is kept open by administration of PGE allowing blood to enter the systemic circulation. Dashed lines indicate the plane of tissue sections shown in d–h. Transverse sections of the muscular DA (d;1 in c), the elastic aorta (e;2 in a), and the ligamentum arteriosum (f;3 in b). Intimal thickening in the DA is indicated in yellow. Areas with CN are indicated in white and the wavy elastic lamellae with blue. Endothelial cells (red outlines) line the inside of the vessels. Longitudinal sections through the DA (4 in a, providing Fig. 1g, and 5 in c providing Fig. 1h). (g) DA tissue and aorta tissue are merging and can be separately distinguished by their characteristic histology. (h) In coarctation, inner media and intimal thickening of the DA extend into the aorta sometimes almost encircling the lumen of the aortic arch at that site (as depicted). The aortic tissue will always be present in the roof of the coarctation.

The fetal ductus arteriosus (DA) is a muscular artery connecting the aortic arch and pulmonary trunk (Fig. 1a). The DA shunts deoxygenated blood from the fetal right ventricle into the umbilico-placental circulation, where gaseous exchange takes place. Prenatally, the DA is indispensable. After birth, as blood becomes oxygenated in the lungs, the DA rapidly contracts followed by anatomical definitive closure (Fig. 1a, b,f). The histological features of the normal closing muscular DA as well as the elastic structure of the aorta are depicted schematically (Fig. 1d,e). DA closure is incomplete in about 10% of neonates [1] causing a patent or persistent DA (PDA). This is the third most common congenital heart defect [2]. Normal closure of the DA, which can take place in both healthy neonates as well as in neonates with congenital heart malformations, is mediated by two processes, vascular smooth muscle cell (VSMC) contraction, and anatomical closure on the basis of tissue degeneration [1], [3]–[5]. Contraction of VSMCs in the DA results from the rapid rise in oxygen tension after delivery [6]. Anatomical closure in humans starts already in the second trimester fetus by the initiation of intimal thickening which is regulated by the prostaglandin (PGE) receptor, EP4 [7] via the protein kinase A and Epac regulatory pathways [8]. After birth following contraction there are major changes in the inner media characterized by extracellular matrix deposition followed by apoptosis and cytolytic necrosis (CN) (Fig. 1d). Eventually a DA ligament (Fig. 1f) is formed [3]–[5], [9]. The molecular regulation of DA regression in the neonate is still incompletely understood.

The anatomical process of postnatal DA closure and regression is common in mammals but the timing of the processes regulating DA closure varies between species. In humans DA maturation already starts in the prenatal period but without strict correlation between gestational age, postnatal age and the degree of maturation [1]. For medical purposes, it is crucial to study the spatiotemporal regulation of DA regression in the prenatal and postnatal period [3]–[5]. In neonates with ductus-dependent heart anomalies, systemic administration of PGE can prevent constriction of the DA [10]. Unrestricted flow via an open DA is essential for those neonates until they can undergo corrective surgery. Histological characteristics of the DA of healthy neonates and those with heart anomalies are identical [3] and similarly influenced by PGE [11]. This offers a unique opportunity to study DA remodeling using surgical biopsy specimens.

Children with Hutchinson Gilford Progeria Syndrome (HGPS) develop progressive arterial occlusive disease and will eventually die due to severe atherosclerosis, see [12]. HGPS is caused by a mutation in *LMNA*, which leads to accumulation of a truncated lamin A protein, progerin, at the nuclear lamina [13]. Arteries of HGPS patients exhibit intimal thickening with dense fibrosis and thinning of the adjacent media [14]. Relative to lesions in typical atherosclerosis, the atheromatous core tends to be smaller [14] or even absent [15] in HGPS arteries. Reduced numbers of VSMCs in the aortic media was a prominent finding in a murine HGPS model [12]. Marked adventitial fibrosis in arteries and veins has been added to the spectrum of vascular lesions in HGPS [14].

The anatomical remodeling process that converts the muscular DA vessel of the fetus and neonate into the fibrous structure of the ligamentum arteriosum is characterized by intimal thickening without an atheromatous core. Furthermore, loss of VSMC in the inner media and intima are due to CN [4], [5], [9]. Although degeneration during closure of the DA and vascular pathology in HGPS is not identical, the similarities in intimal thickening and loss of VCMCs warrant the hypothesis that progerin may also play a role in the DA closure. Here, we show that progerin is specifically expressed during the closing process of the DA in areas with developing CN, while Lamin A/C is hardly expressed in these regions. The molecular and cellular implications of these novel findings are discussed.

## Results

**Figure 2:**
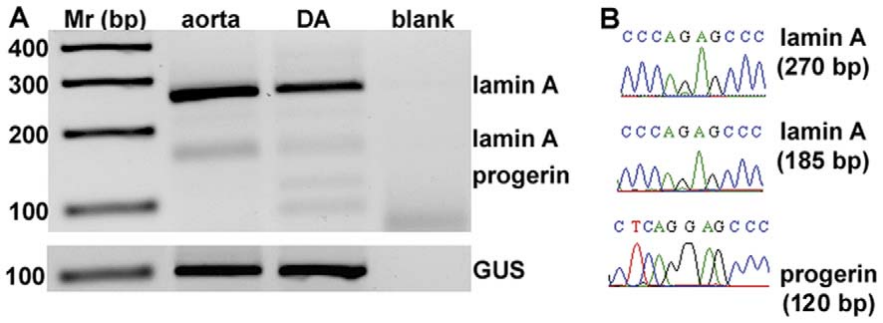
RT-PCR reveals expression of progerin mRNA in DA. **A.** RT-PCR analysis of *LMNA* and progerin mRNA in neonatal aorta and DA was performed with primers that amplify 270 and 185 bp of *LMNA*, and 120 bp of progerin. GUSB PCR product was used as a control. Aorta tissue was obtained from a 4-month patient and DA tissues from 6 neonates 8 days after birth. **B.** Direct sequencing of PCR fragments 270, 185 and 120 bp. Shown are the sequence histograms from position 2212 bp.

To determine whether progerin is expressed in neonatal DA, reverse transcriptase (RT) PCR was performed on fresh and frozen biopsies of DA that were collected during corrective cardiac surgery. The expression of progerin mRNA was analyzed in neonatal DA sections using primers that cover the progerin cryptic site [16]. The full length mRNA PCR product is 270 bp (Fig. 2A). In addition a 185 bp product representing the delta 10 isoform was amplified in the DA and aorta tissues. Importantly, in the neonatal DA but not in aorta tissue a 120 bp PCR product was found (Fig. 2A). These PCR products were evaluated by sequencing analysis which confirmed that the 120 bp fragment corresponds to progerin whereas the 270 bp fragment is of *LMNA* full length mRNA (Fig. 2B). It is relevant to note that in the analyzed biopsies this primer set led to unspecific PCR products (i.e. a 100 bp fragment; Fig. 2A). Therefore, it was essential to confirm *LMNA* gene products by sequencing of the PCR products. The housekeeping beta glucuronidase (GUSB) gene was used as a control [17] and PCR product was found in all tissues. In total, progerin PCR product was found in all 6 DA samples analyzed, but was not detected in the two aorta samples. Aorta and DA are physically connected, while remodeling in neonates is limited to the DA, suggesting an association between progerin and DA remodeling.

**Figure 3:**
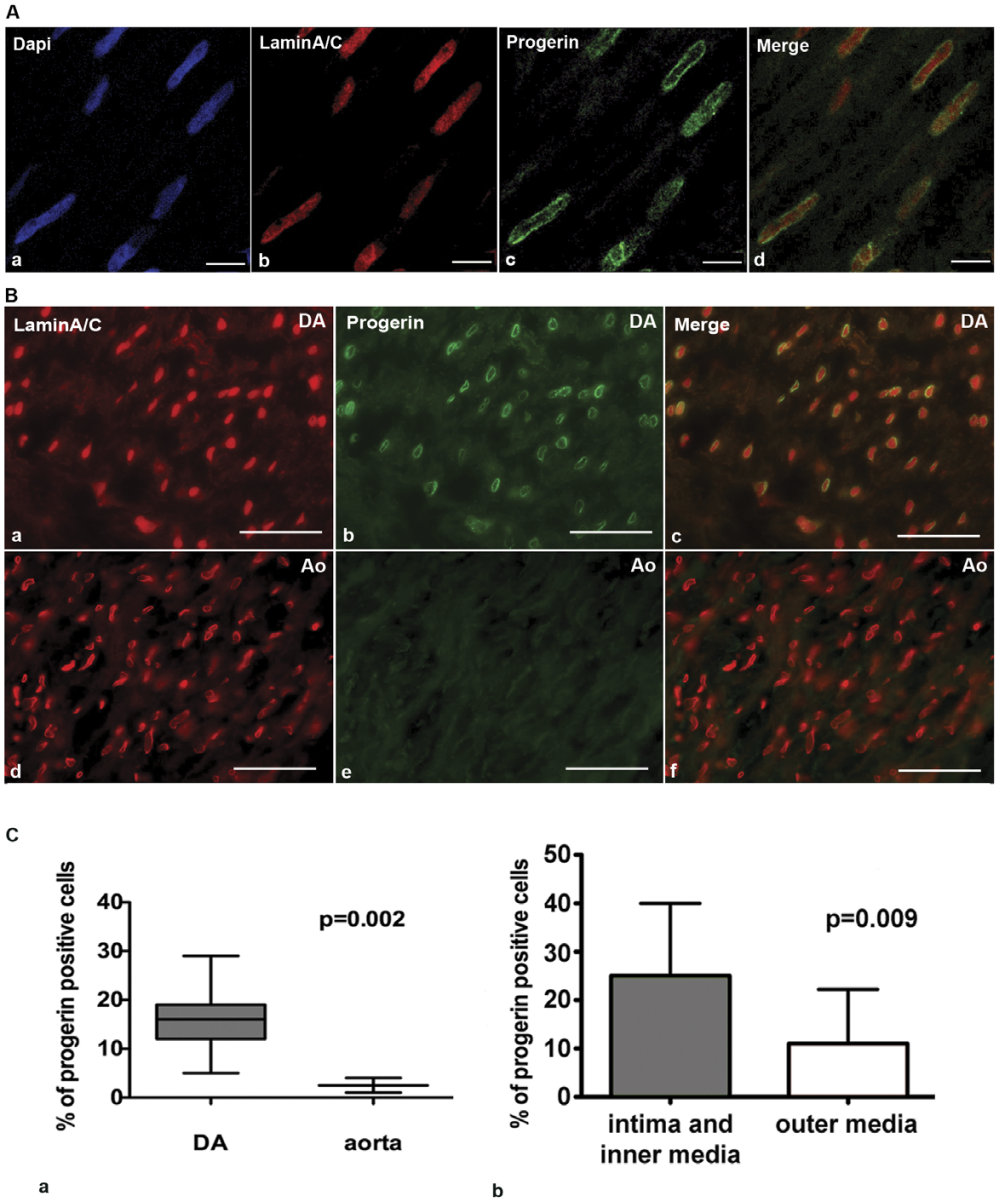
Spatial changes of progerin expression in a neonatal DA. **A.** (b) Confocal images of immunofluorescence show even nuclear distribution of lamin A/C (red) and (c) nuclear envelope localization of progerin (green) in the media of DA. (a) Nuclei are counterstained with DAPI. (d) Merging of the staining patterns. Scale bars: 5 µm. **B.** Microscopic image of immunofluorescence in DA and aorta (Ao) show (a,d) the tissue specific expression of lamin A/C and (b,e) progerin. (c,f) in the media. Nuclei expressing both proteins as shown in the merged images. Scale bars: 20 µm. **C.** (a) Box-plot shows the percentage of progerin positive cells in DA and aorta. Statistical analysis represents 1302 nuclei in DA and 302 nuclei in aortas from 5 individuals. P-value indicates significant difference in progerin expression between DA and aorta. (b) Histograms show the average ratio of progerin positive cells in intima and inner media *versus* the outer media. Statistical analysis represents 4 samples from each of the 5 individuals. P-value indicates that intima and inner media contain significantly more progerin expressing cells than the outer media.

The low level of progerin mRNA in DA indicates that the protein would be expressed at very low levels or in only part of the cells. Progerin and lamin A/C protein expression was analyzed with immunohistochemistry using antibodies that specifically recognize progerin or lamin A/C [16]. Due to irreversible farnesylation at the C-terminus, progerin accumulates at the nuclear lamina [18]. In contrast, lamin A/C is also found in the nucleoplasm [18]. Indeed, confocal microscopy revealed progerin expression (Figure 3A,c), at the nuclear lamina, while lamin A/C exhibited an additional nucleoplasm distribution (Fig. 3A,b). This confirms that the two antibodies recognize different proteins.

**Figure 4:**
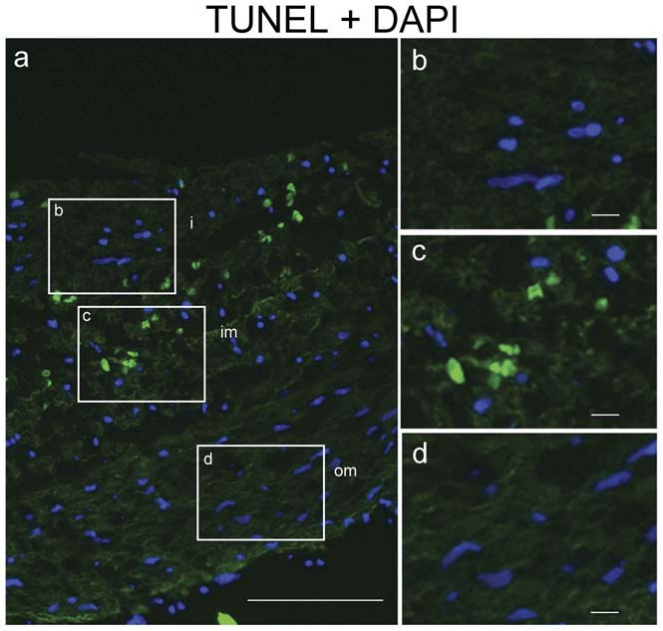
Spatial distribution of TUNEL staining in the DA. (a) A sister section of the neonatal DA from figure 3A that was stained with TUNEL (green). Details of the staining are shown in magnifications of the boxed areas b–d. TUNEL staining is predominantly found in the inner media (c) and the bordering intima (b). TUNEL positive nuclei were not found in the outer media (d). Nuclei were counter stained with DAPI. Scale bars are: scale bar 100 µm (a); 10 µm (b,c,d).

Progerin expression in neonatal DA was found in a large number of cells, while in the aorta its expression was less prominent (Fig. 3B b,e). We determined that the mean fraction of progerin expressing cells in the DA was 16.2%±7.2 while in the aorta it was only 2.5%±1.5 (Fig. 3C,a) indicative for a tissue-specific expression of progerin. Since the major histological difference between the DA and aorta in neonates is degeneration of the inner media and the intima, we determined progerin expression across the DA layers. A higher fraction of progerin positive cells was found in the intima and inner media (25%±15.0), compared to the outer media (11%±11.2) (Fig. 3C,b). This difference in the spatial distribution of progerin was statistically significant. TUNEL staining of the neonatal DA, indicative of apoptosis, was predominantly found in the inner media bordering the intima (Fig. 4a–d). As cytolytic necrosis will initially develop in cells of the intima and inner media, the high expression of progerin in these layers supports a functional relationship with cell death. The spatial overlap in the DA between progerin expression and TUNEL, suggests a causal relation between progerin expression and apoptosis in the development of CN.

**Figure 5:**
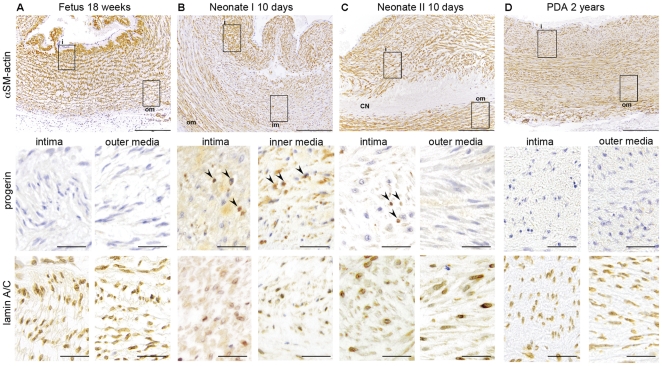
Spatial and temporal expression of progerin is associated with development of CN in the DA. A. Overview of the wall of the DA of an 18-week-old fetus depicting α-smooth muscle actin (αSM actin) staining of the vascular smooth muscle cells. Details of intima (i) and outer media (om) show the absence of progerin and the overall expression of lamin A/C in the cells. B. Overview of the DA of a 10-day-old neonate with onset of degeneration of the inner media (im). In this layer there is loss of α-SM actin staining. Details of intima and inner media show progerin expressing nuclei (arrowheads) and reduction of lamin A/C expressing cells compared to the fetal DA in A. C. Overview of the DA of a 10-day-old neonate with marked cytolytic necrosis (CN) in the inner media with loss of cells. Smooth muscle cells are still detectable in the intima and outer media. Detail of the intima shows some progerin expressing nuclei (arrowheads) and absence of these nuclei in the outer media. Lamin A/C expression is found in the inner and outer media. D. Overview of a non-closing persistent DA (PDA) of a 2-year-old infant. In all layers there is abundant α-SM actin expression. Details of the intima and outer media show absence of progerin and presence of lamin A/C expressing cells. Scale bars: overviews A–D 200 µm, details 50 µm.

Progerin expression was analyzed in 16 neonatal DA biopsies (including 2 with adjacent aortic tissue) with various degrees of CN development. Furthermore, specimen from a 14- and an 18-week-old fetus and a PDA from a 2-year-old child were studied. In the fetal DA CN is not developed, and, as expected, uniform SM-actin staining was found across the DA layers (Fig. 5A). Regression of the normal neonatal closing DA is characterized by a reduced expression of contractile proteins, such as alpha-SM-actin in the inner media [5], [9]. In the DA from neonate I (Fig. 5B) SM-actin expression in the inner media was less intense compared to the adjacent wall layers. In the same area from the 10-day-old infant II (Fig. 5C), however, prominent CN in the inner media was present showing a zone without cells and complete absence of alpha-SM-actin expression (Fig. 5C). These represent two subsequent stages in the degeneration of the neonatal DA. Spatially unchanged SM-actin staining was exhibited in the PDA (Fig. 5D) similar to the fetal DA. In both the fetal and PDA without CN, progerin expression was not detected (Figure 5A,D). In contrast, in the DA of both neonates progerin expression was found in the intima and inner media but not in the outer media (Fig. 5B,C), where VSMCs remain present throughout ligament formation.

Since progerin is a splicing variant of the lamin A gene, we investigated the expression pattern of lamin A/C in the DA. Lamin A/C expression was found in all nuclei in the fetal DA (Fig. 5A), but in neonatal DA the expression was markedly less in areas of developing CN (Fig. 5B,C). Lamin A/C was found in all layers in the PDA (Fig. 5D) indicating a spatiotemporal association between lamin A/C expression and the development of CN in the normal closing neonatal DA.

## Discussion

The molecular mechanisms that are involved in DA closure are poorly understood. Here we report, for the first time, progerin expression in the neonatal DA. We demonstrated expression of progerin predominantly in the neonatal but not in the fetal DA, and in a higher number of cells in intima and inner media, compared to the outer media and to the aorta. This spatial and temporal progerin expression pattern highly correlates with CN development in the degenerating neonatal DA implying a functional role for progerin in CN development. Abnormal DA closure was not reported so far in HGPS patients or in the HGPS mouse models. To confirm a role for progerin in DA closure detailed studies in neonatal HGPS models and patients are relevant.

Cell death in the DA is accompanied by activation of apoptosis [9], [19]. We show a spatial correlation between progerin expression and frequency of apoptosis. We postulate that progerin plays a role in cell death of smooth muscle cells in the DA. Reduction of alpha-SM-actin was found in arteries of HGPS patients in cells expressing progerin [13] indicating that spatial and temporal expression of progerin correlates with development of apoptosis. Apoptosis frequency is increased in fibroblasts from HGPS [20] and elevated apoptosis in the mouse model for progeria is reported [12]. The role of progerin in initiating apoptosis of cells in the inner media and intima of the DA should be further investigated.

Elevated progerin expression was spatially associated with reduced lamin A/C expression in the DA. These spatial changes between lamin A/C and progerin expression in the inner media and intima suggest an association with alternative splicing of *LMNA* gene products during DA remodeling. Activation of *LMNA* alternative splicing was found during cell differentiation and cell cycle progression [21]. During postnatal cardiac development splicing variants of *ASF/SF2* and *SERCA2* are activated [22], [23] to change cellular function. Lamin A/C was recently shown to play a role in cardiac disease. In heterozygous *LMNA*+/− knockout mice, a 50% reduction in lamin A/C protein levels cause myocardial apoptosis and cardiac abnormalities [24]. Deregulation of lamin A/C processing correlates with atherosclerotic lesions from aged individuals Pre-lamin A accumulates in medial VSMC from aged individuals and in atherosclerotic lesions correlating with the down-regulation of lamin A/C processing enzymes [25]. Based on these studies we hypothesize that changes in lamin A/C expression and activation of splicing variants are an alternative mechanism to regulate cardiovascular development. Alternative splicing is affected by oxidative stress [26]. Whether oxygen levels alter *LMNA* splicing during DA closure is yet to be determined Anatomical definitive sealing of the DA is preceded by contraction of VSMCs in which many molecular pathways are involved [1] e.g. activated by reactive oxygen species. Accumulation of reactive oxygen species has been described in both the onset of DA closure [27] as well as in vascular ageing [28]. Progerin expression in coronary arteries of non-HGPS individuals, which significantly increases throughout life has been associated with vascular ageing [14]. Farnesyltransferase inhibitors (FTIs) can in part correct cellular defects caused by progerin [29], [30]. FTIs reverse the permanent farnesylation of progerin or pre-lamin A and, thus repair structural and functional nuclear defects. Given our finding that progerin is expressed in the neonatal DA, it will be relevant to study whether FTIs can interfere with local vascular remodeling and prevent anatomical DA closure.

Our study indicates a novel role for progerin in a programmed cellular process although a relation between DA closure and the process of ageing needs further clarification.

## Materials and Methods

### Ethics statement

The medical ethics committee of the Leiden University Medical Center exempted this study from review because tissues normally discarded were analyzed anonymously and waived the need for consent due to the fact that linkage of the samples received with individual patients was not possible.

### Tissue samples

Postnatal human DA tissues were obtained during surgical correction of cardiac anomalies. The resection of the DA was part of the surgical strategy for aortic arch reconstruction in all cases (see Figs. 1c,h, and dashed line 5 for site and orientation of the dissected biopsies). Fetal tissues were acquired from post-mortem specimens after legal or spontaneous abortion after PGE induction. All tissues were collected and stored at the department of Anatomy and Embryology of Leiden University. Tissue type, diagnosis and age were recorded for this study.

Tissue samples were directly fixed in 4% phosphate-buffered formaldehyde or 2% acetic acid in absolute ethanol at room temperature. Fixed samples were embedded in paraffin and sectioned at 5 µm. For RNA extraction or cryostat sections, samples were placed in Tissue-tek, and were directly frozen in liquid nitrogen and stored at minimal −20 C. Fetal material was obtained from 2 cases (14 and 18 weeks gestation) providing aorta and DA wall tissue. Neonatal tissue was studied in 16 DA biopsies and two descending aortas. The neonates (aged 3–21 days, median 10 days) were born after 38±2 weeks of gestation. All of them received a low dose of PGE (0,0125 µg/kg/min) because of left-sided cardiac defects with aortic arch anomalies (Fig. 1 c,h, dashed line 5). The biopsy of a PDA came from a 2-years-old child. A part of the descending aorta was collected from a 4-month-old infant during coarctation repair and served as a negative control (not shown).

### Immunohistochemistry microscopy and image quantification

For a morphological overview sections were briefly (5 seconds) counter stained with haematoxylin eosin. Antibodies used in this study are: monoclonal lamin A/C (Chemicon International (Temecula, CA, USA); 1∶50) 1∶50, a monoclonal anti-lamin A G608 (progerin) antibody, kindly provided by K. Djabali (Dpt. of Dermatology, Columbia University, NY, USA) and mouse monoclonal antibody against smooth muscle alpha-actin 1A4 (1∶3000; DAKO A/S Glostrup, Denmark). Antibody incubation was performed in PBS containing 0.05% Tween 20 (PBS-T) and 1% bovine serum albumin overnight in a humidified chamber with the primary antibody at room temperature. The first antibody was detected with either HRP-conjugated secondary antibody (Vector Laboratories, Inc. Burlingame, CA, USA) or with a fluorescent- conjugated secondary antibody (Goat-anti-mouse/Alex 488, Invitrogen, Corp. Carlsbad, CA, USA, Donkey-anti-mouse/Cy3, Jackson ImmunoResearch Labaratories, West Grove, PA, USA). The fluorescent conjugated secondary were mounted in Citifluor (Agar Scientific) containing 400 µg/ml DAPI (Sigma-Aldrich) and examined with a confocal laser scanning microscopy (Leica SP5) or a Fluorescence microscope (Leica DM RXA), 63× and 100× lens NA 1.4 plan Apo objective. Apoptotic cells were determined using the in situ Cell Death Detection Fluorescein kit from Roche Diagnostics (Almere, Nederland) following the manufacturer's instructions. Significance of statistical analysis was performed with the unpaired T-test with Welch's correction.

### RNA isolation and RT-PCR reaction

Total RNA was extracted from frozen biopsies using the RNA-Bee RNA extraction kit as per the manufacturer (AMS Biotechnology, Abingdon, U.K.). 1 µg of RNA from each sample was submitted to cDNA synthesis using the RevertAid™ First Strand cDNA Synthesis Kit (Fermantas, Ontario, Canada) or with the SuperScript® VILO™ cDNA Synthesis Kit (Invitrogen). 1 3.4 ng of cDNA was used for PCR using primers located in exon 9 and 12 of *LMNA* (forward: 5′ ACCCCGCTGAGTACAACCT, and reverse: 5′ TGCAGTTCTGGGGGCTCT. The primers cover progerin cryptic site at position 2215 in *LMNA* mRNA, and are 2 bp longer than these that were previously described. Our set of primers gives less non-*LMNA* PCR products in DA and aorta as compared those reported in [31]. PCR conditions were 35 cycles, each cycle consisting of 30-sec denaturation at 94°C, 30-sec annealing at 60°C, and 30-sec polymerization at 72°C. 15 µl of the reaction was analyzed on 2% agarose gel and stained with ethidium bromide. PCR products were eluted from gel and were sequenced at www.lgtc.nl. Primers for GUSB amplification are: forward- 5′CTCATTTGGAATTTTGCCGATT 3′; reverse- CCGAGTGAAGATCCCCTTTTTA.
