# Correction: Differential Temporal and Spatial Progerin Expression during Closure of the Ductus Arteriosus in Neonates

**DOI:** 10.1371/annotation/c0198e87-d087-444c-8be9-780adb1582be

**Published:** 2011-09-23

**Authors:** Regina Bökenkamp, Vered Raz, Andrea Venema, Marco C. DeRuiter, Conny van Munsteren, Michelle Olive, Elizabeth G. Nabel, Adriana C. Gittenberger-de Groot

The funding information is incorrect. These authors have no support or funding to report. 

